# Defining the Low End of Primate Social Complexity: The Social Organization of the Nocturnal White-Footed Sportive Lemur (*Lepilemur leucopus*)

**DOI:** 10.1007/s10764-013-9735-3

**Published:** 2013-11-16

**Authors:** Iris Dröscher, Peter M. Kappeler

**Affiliations:** 1 Behavioral Ecology & Sociobiology Unit, German Primate Center, 37077 Göttingen, Germany; 2 Department of Sociobiology/Anthropology, Johann-Friedrich-Blumenbach Institute of Zoology & Anthropology, University of Göttingen, 37077 Göttingen, Germany

**Keywords:** Cohesiveness, Dispersed pairs, *Lepilemur*, Pair-living, Social complexity

## Abstract

Whereas other species of sportive lemurs (genus *Lepilemur*) have been described as living in dispersed pairs, which are characterized by spatial overlap but a lack of affinity or affiliation between one adult male and female, existing reports on the social organization of the white-footed sportive lemur (*Lepilemur leucopus*) are conflicting, describing them as either living in dispersed one-male multifemale systems or pairs. We conducted this study in the spiny forest of Berenty Reserve, southern Madagascar, to clarify the social organization and to characterize the level of social complexity of this species. We combined 1530 h of radio-telemetry and behavioral observations over a period of 1 yr to describe the spatiotemporal stability, size, and interindividual overlap of individual home ranges as well as interindividual cohesiveness. Results revealed low intra- and high intersexual home range overlap. Although most of the social units identified consisted of dispersed pairs (*N* = 5), males were associated with two adult females in two cases. Furthermore, members of a social unit were never observed to groom each other or to share a daytime sleeping site, and Hutchinson’s and Doncaster’s dynamic interaction tests indicated active avoidance between pair partners. Low cohesiveness together with extremely low rates of social interactions therefore arguably places *Lepilemur leucopus* at the low end of primate social complexity.

## Introduction

The majority of primate field studies have been concerned with descriptions and analyses of their social systems (Crook and Gartlan 1966; Mitani *et al.* 2012; Smuts *et al.* 1987). There is an emerging consensus that the diversity of primate social systems can be analyzed at the level of their social organization, mating system and social structure (Cords 2007; Kappeler and van Schaik 2002; Kappeler *et al*. 2013). Interspecific variation in social systems has also been analyzed more recently as a function of social complexity (Bergman 2010; de Waal and Tyack 2003; Lehmann and Ross 2011; McComb and Semple 2005). In this context, complex social systems have been defined as those in which individuals frequently interact in many different contexts with many different individuals, and often repeatedly interact with many of the same individuals in networks over time (Freeberg *et al*. 2012). Social complexity is therefore an integrative measure that correlates positively with group size because, according to the social intelligence hypothesis (Dunbar 1998), animals living in larger groups should have enhanced cognitive abilities to facilitate the management of multiple social relationships, compared to those living in smaller groups or in other types of social organization (Bond *et al*. 2003; Byrne and Whiten 1988; Dunbar and Shultz 2007). However, social complexity has not been explicitly studied in primates that do not live in groups, so that it is currently difficult to define a baseline for comparative studies of social complexity.

The absence of group living among primates correlates strongly with nocturnal activity (van Schaik 1983). Even though about a quarter of all primates are nocturnal, their social systems have remained comparatively poorly studied (Bearder 1999). Nocturnal primates have initially been collectively characterized as “solitary foragers” (Bearder 1987; Petter *et al*. 1977), but methodological advances in telemetry and molecular genetics have since disclosed more details of the diversity and complexity of their social systems (summarized in Kappeler 2012; Nekaris and Bearder 2011). In particular, some species of dwarf (*Cheirogaleus* spp.) and sportive lemurs (*Lepilemur* spp.) were found to be organized into pairs, even though individuals forage solitarily (Fietz 1999; Müller 1999; Rasoloharijaona *et al.* 2003; Zinner *et al*. 2003). Because pairs are the smallest social units, and pair-living requires active coordination between pair partners (Barelli *et al*. 2008; Schülke and Kappeler 2003), and because its evolutionary emergence was associated with a significant increase in brain size (Shultz and Dunbar 2007), pairs represent an interesting level of analysis for comparative studies of social complexity. Species that combine aspects of the likely evolutionary transition between a solitary social organization and pair living might be particularly interesting in this context because they may represent the earliest and most primitive form of sociality.

Our study focused on a species of sportive lemur for which conflicting information about the social organization of the same population had been reported. White-footed sportive lemurs (*Lepilemur leucopus*) are confined to the region between the Menarandra and Mandrare Rivers in southern Madagascar (Hoffmann 2008). They have evolved adaptations to a folivorous diet despite small body size (<1 kg), including prolonged resting bouts, small night ranges, a prolonged cecum, and cecotrophy (Hladik and Charles-Dominique 1974). Two short field studies were conducted on the same population of white-footed sportive lemurs at Berenty Reserve in the 1970s and reported conflicting patterns of social organization (Charles-Dominique and Hladik 1971; Russell 1977). Charles-Dominique and Hladik (1971) described exclusive range use by both sexes, but range overlap between the sexes, and found that the largest male was associated with five females. Russell (1977) reported that no individual had an exclusive range and described range-sharing by females. He also observed males and females sleeping together during the day. Based on these observations, the social organization of *Lepilemur leucopus* has been classified as a “dispersed harem” (Müller and Thalmann 2000). Neither study used radio-tracking or detailed patterns of social interactions.

The present study aimed to resolve these conflicting reports by characterizing the social organization of *Lepilemur leucopus* during a year-long study of radio-collared individuals. In particular, we empirically identified natural social units and investigated their stability across the year. In addition, we quantified the degree of cohesiveness within social units using three different computational approaches and report on patterns of social interactions within and between social units. Together, these data also contribute to our second aim, namely the characterization of the level of social complexity in this small nocturnal lemur.

## Methods

### Study Site and Subjects

We conducted this study at Berenty (S 25.00°, E 46.30°), a *ca.* 200 km^2^ private ecotourism reserve located in southern Madagascar. To ensure continuing focal observations of single individuals throughout the night, we equipped subjects with radio-tracking transmitters. We captured the individuals by blow-darting in a spiny forest fragment of *ca.* 5 ha (HAH Reserve Forestière parcel 1), which is connected to gallery forest on one side via a transitional forest and a further 40-ha spiny forest fragment on the other side (Norscia and Palagi 2008).

We used a blowpipe and 1-ml air pressured narcotic syringe projectiles (Telinject, Germany) to anesthetize subjects with 0.4 ml of Ketanest (100 mg/L) in the mornings in their daytime resting sites. We captured anesthetized individuals with a blanket when they fell out of the tree. Alternatively, if the anesthetized individuals did not fall and it was possible to reach them by climbing the tree, we retrieved them from their resting sites by hand or with an animal capture pole (Tomahawk 7′–12′ extension restraint pole). We fitted the subjects with radio-collars (TW-3 button-cell tags, Biotrack, U.K.) while anesthetized. We kept the subjects in an animal transport box (Traveller Box Capri Mini, Trixie Heimtierbedarf, 40 × 22 × 30 cm) until they were fully recovered and released them again at their capture site in the evening. The same individuals later reused sleeping trees where they were captured.

We fitted 16 adult (8 males and 8 females) and 4 subadult individuals (3 males and 1 female) with radio-collars. We differentiated adult individuals from subadults by the degree of tooth wear and body mass. At the beginning of the study, all subadult individuals still ranged within their parental territories. Once they dispersed from their natal range, we classified them as adults. We did not radio-collar smaller juvenile individuals because radio-collars exceeded 4% of their body mass. Some members of social units were not equipped with radio-collars. However, we noted their presence during capture of subjects, focal animal observations, and a population census at the end of the study. We removed all radio-collars after the end of the study. The research followed standard protocols for animal handling, capture, and radio-tracking and was approved by the Commission Tripartite CAFF (Madagascar).

### Behavioral Observations

We collected behavioral and locational data between October 2011 and October 2012 for a total of 1530 h on 20 radio-collared individuals. We divided the study period into four biologically relevant seasons: birth and offspring care with lactation (early wet season from November to January), offspring care without lactation (late wet season from February to April), mating and early gestation (early dry season from May to July), and late gestation (late wet season from August to October).We followed each radio-collared individual for up to 2 full nights during each season, with a TR-4 receiver and a RA-14K antenna (Telonics, U.S.A.).The number of focal animal follows per season decreased throughout the year owing to the disappearance of individuals, so that the total number of focal animal follows per individual ranged between 5 and 8 nights (mean ± SD: 7.7 ± 0.8 nights per individual). We restricted our analyses of static and dynamic spatial interactions to adult individuals belonging to seven different social units (Table I).

**Table I:** Summary of continuous focal animal observations conducted throughout the year

Social unit	Male ID	Hours	*N* location points	Female ID	Hours	*N* location points
1	m10	57	642	f1B	87	990
2	m9	79	863	f2	88	996
3	m3	88	946	f3	88	964
4	m4	90	1008	f4	86	991
5	m5	88	971	f5	88	994
6	m6	87	948	f6	90	1010
7	m7	89	1012	f7	87	988

The trees of the spiny forest have small and exposed canopies (Grubb 2003), permitting observation of the subjects clearly and continuously, despite their nocturnal activity (Hladik and Charles-Dominique 1974). Continuous focal animal observations (Altmann 1974) started when an individual left its sleeping site at dusk and were continued until it returned to its daytime resting tree at dawn. On average the focal individuals were out of sight for 7.1 ± 1.8% (mean ± SD) of total observation time. We identified individuals ranging in the same area before the onset of data collection during preliminary observations on sleeping site choice and ranging behavior of radio-collared individuals. Henceforth, a second trained observer followed the range mate of a focal individual simultaneously. We tagged spatial locations of subjcts during continuous focal observations with biodegradable tape. After each full-night follow, we determined the exact position of the tagged trees with reference to a 10 × 10 m study grid system. Each morning after a full-night follow we located the sleeping trees of all radio-collared individuals by radio-tracking to determine the composition of sleeping associations.

We defined social interactions as agonistic, affiliative, or neutral. We defined all interactions that were either aggressive (chase, charge, bite, and grab) or submissive (flee, be displaced, or jump away) as agonistic (*sensu* Pereira and Kappeler 1997). We noted interactions during which individuals sat ≤1 m of each other and/or groomed each other as affiliative. We termed interactions during which individuals came within a distance of 5 m of each other without exhibiting agonistic or affiliative behavior as neutral. We based calculations of the frequency of social interactions on the time the focal individuals were actually in sight.

### Data Analyses

To evaluate static spatial interactions between subjects, we calculated individual annual home ranges with the Animal Movement extension of ArcView. We subsampled locational data at 5-min intervals for home range analyses. We calculated home range size from 95% fixed kernel home range utilization distributions (Worton 1989) using *ad hoc* smoothing (Silverman 1986). We did not correct for spatial autocorrelation, as kernel densities do not require serial independence of observations when estimating home ranges size, and the accuracy and precision of home range estimates improve with the number of observations (De Solla *et al*. 1999). We calculated home range overlap in R (R Core Team 2012) using the package adehabitatHR (Calenge 2006). To determine whether social units were maintained throughout the year, we calculated overlap of night ranges of simultaneously followed males and females as percent overlap (Kernohan *et al*. 2001). We calculated overlap of annual home ranges for both, pair partners and same-sexed neighbors. We calculated seasonal influence on night range overlap for pair partners that were followed simultaneously, using one-way repeated-measures ANOVA. We excluded one pair (m10fB1) from the analyses because simultaneous follows on the pair partners were conducted only during the wet season. We averaged values for each season and pair. The data were normally distributed for each level of the within-subject factor season. We conducted the analyses in R using the function ezANOVA in the package ez (Lawrence 2012).

We examined dynamic spatial interaction to quantify the degree of sociality between pair partners, i.e., whether they associated, avoided each other, or moved randomly in relation to each other. We used three different models: the random gas model (Waser 1976), Hutchinson’s model (Hutchinson and Waser 2007), and Doncaster’s model (Doncaster 1990). We calculated expected rates of encounters between pair partners with the random gas model as $f=\frac{\left(4\times \rho \times v\right)}{\pi}\times \left(2d+s\right)$, where *ρ* is the density of a species, *υ* the velocity of an individual, *s* the group spread, and *d* the distance criterion. We calculated expected rates of associations between pair partners with Hutchinson’s model as *f* = *N* × *ρ* × *π* × *d* ^2^, where *N* is the number of instantaneous observations, *ρ* is the density of a species, and *d* the distance criterion. For both models, we compared observed rates with expected rates, using Wilcoxon signed-ranks test across all pairs. Using Doncaster’s model, we compared *N* observed interindividual distances with expected ones calculated from all *N* ^2^ distances possible within a given set of spatial points. We compared observed with expected values for each pair within a 2 × 2 contingency table containing counts below and above *d* using a χ^2^ test. The significance test depends on successive data points being independent, giving each individual the opportunity to travel to any other part of its range between successive instantaneous observations (Doncaster 1990). We considered data points to be independent as the interval permits an individual to traverse its home range at maximum travel speed (Rooney *et al.* 1998). Here, we calculated *ρ* as the inverse of a pair’s union home range in square meters and *υ* as the average distance the male and female covered during the observation period in meters. We set *s* to zero and *d* to 15 m as this distance was close enough to allow visual contact between individuals. We used the software R for statistical analyses. We considered α levels of *P* ≤ 0.05 as statistically significant.

## Results

### Static and Dynamic Spatial Interactions

**Fig. 1:**
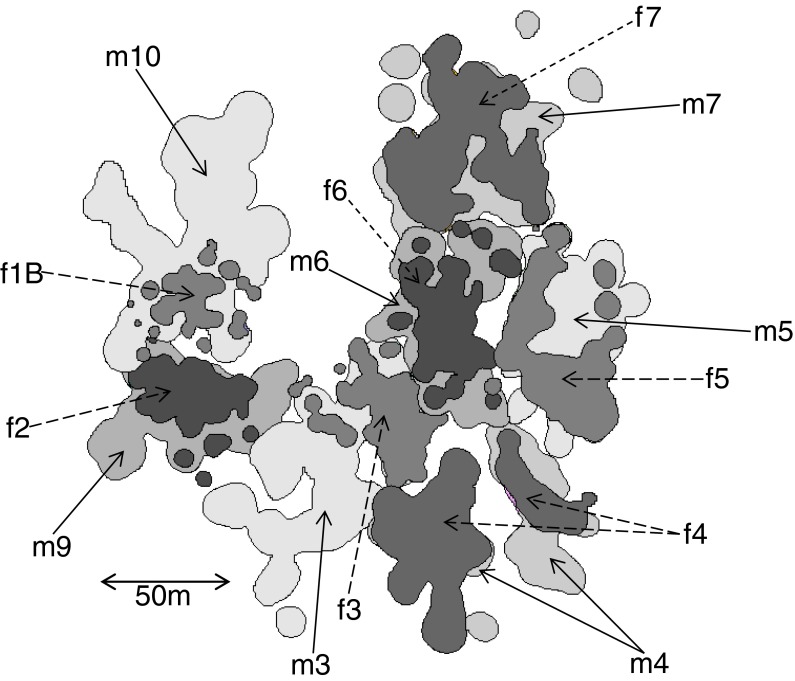
Annual home ranges of adult males (m) and females (f) of *L. leucopus*. Depicted are the 95% kernel home ranges of each individual.

Average annual home ranges were significantly larger for males (mean ± SD: 0.33 ± 0.08 ha, *N* = 7) than females (0.18 ± 0.08 ha, *N* = 7; Wilcoxon rank sum test: *W* = 47, *N* = 14, *P* = 0.005; Fig. 1). Male annual home ranges overlapped on average with those of neighboring males by only 1.65 ± 1.99% and those of females with those of neighboring females by merely 0.4 ± 0.64% (mean ± SD) based on nine dyads of possible neighbor pairings. However, annual home ranges of particular males and females overlapped considerably. Average overlap between the annual home ranges of the seven pairs identified was 81 ± 20% for females and 43 ± 16% (mean ± SD) for males. Differences between male and female’s perspective are due to the smaller home ranges of females.

Average overlap of night ranges was 73 ± 28% for females and 45 ± 24% (mean ± SD) for males based on six identified pairs. In general, overlap between pair partners was high throughout all seasons. The maximum observed night range overlap was 100% from the female’s perspective and 93% from the male’s perspective. Only during 2 out of 48 simultaneous follows did night ranges of pair partners not overlap. Otherwise, minimum observed night range overlap was 26% from the female’s perspective and 12% from the male’s perspective. Furthermore, season did not have a significant effect on night range overlap from the female’s perspective (one-way repeated-measures ANOVA: *F*_3,5_ = 1.25, *P* = 0.33). However, season had an influence on night range overlap from the male’s perspective (one-way repeated-measures ANOVA: *F*_3,5_ = 3.26, *P* = 0.05). Night range overlap of males with their corresponding female pair partners was significantly higher during the early dry season (corresponding to mating and early gestation) compared to the late dry season (corresponding to late gestation; Tukey’s *post hoc* test: *Z* = –3.30, *P* = 0.005).

**Fig. 2:**
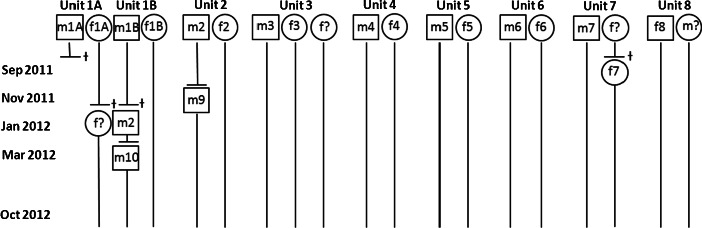
Demographic changes in the study population between September 2011 and October 2012. Only adult animals are presented. Males are represented by squares; females by circles. Confirmed deaths are illustrated with a cross. No ranging data are available for individuals labeled with a question mark as they were not equipped with radio-collars or died before they could be followed.

Static spatial interactions between adult individuals changed during the course of the study due to confirmed deaths and dispersal events (Fig. 2). Demographic changes took place within social units 1, 2, and 7. The home range of male m3 overlapped with those of two females from the beginning of the study, whereas male m10 ranged with two females from March 2012 onwards.

Based on the random gas model, observed encounter rates between pair partners were significantly higher than expected (Wilcoxon signed rank test: *V* = 28, *N* = 7, *P* = 0.02). In contrast, using Hutchinson’s model, the number of observed associations was significantly lower than expected (Wilcoxon signed rank test: *V* = 0, *N* = 7, *P* = 0.02). Similarly, observed values were significantly smaller than expected for five out of seven pairs (χ^2^ ≥ 3.87, df = 1, *P* ≤ 0.049) and nonsignificant for the remaining two pairs (χ^2^ ≤ 0.7, df = 1, *P* ≥ 0.28) using Doncaster’s model.

### Sleeping Associations

The focal individuals spent the day mainly in the confluence of branches of trees of the genus *Alluaudia*, or more rarely inside liana tangles or tree holes. A single adult used 5–11 different sleeping sites (Table II). Although adult individuals shared some of their sleeping trees with their pair partner, they never used them simultaneously (37–69 observation days per dyad). In contrast, adult females and their offspring shared sleeping trees during 79% of 42 observation days, based on six adult females that gave birth in November 2011. However, we never saw adult males sharing a sleeping tree simultaneously with any other member of their social unit. Sleeping trees were never shared with neighbors, neither simultaneously nor consecutively.

**Table II:** Number of sleeping trees used exclusively, shared with pair partner and days of simultaneous use

Pair	Observation days	Exclusive use m/f	Shared use	Days simultaneous use
m10f1B	37	3/3	3	0
m9f2	61	8/4	1	0
m3f3	69	9/3	2	0
m4f4	69	8/4	2	0
m5f5	69	5/4	1	0
m6f6	69	1/2	3	0
m7f7	69	7/2	3	0

### Social Interactions

In total, we observed 72 social interactions during 516 h of simultaneous focal observations on range mates. The frequency of observed social interactions was therefore low, with only 0.14 interactions/h across all pairs (Table III). Most social interactions were of the neutral type (78%), i.e., individuals sitting 1–5 m of each other. Agonistic interactions were less common than neutral interactions (21%), whereas affiliative interactions were essentially absent (1%). We never observed allogrooming between adult males and females or social interactions between neighboring males. We only observed a single affiliative social interaction between neighboring adult females (f1B and f2). We did not observe any social interactions between the females of the two social units (1 and 3) with two adult females. However, we observed agonistic interactions between resident and floating males. We did not witness any matings.

**Table III:** Frequency and types of social interactions between pair partners

Pair	Neutral	Agonistic	Affiliative	Total
m10f1B	0.12	0.02	0.00	0.15
m9f2	0.24	0.03	0.01	0.28
m3f3	0.09	0.04	0.00	0.13
m4f4	0.07	0.02	0.00	0.10
m5f5	0.05	0.00	0.00	0.05
m6f6	0.12	0.05	0.00	0.17
m7f7	0.07	0.05	0.00	0.12
Mean	0.11 ± 0.06	0.03 ± 0.02	0.00 ± 0.01	0.14 ± 0.07

Total number of observation hours: 516.

## Discussion

### Static Spatial Interactions

Adult white-footed sportive lemurs had almost exclusive home ranges, as range overlap among neighboring adult males (1.7%) as well as among neighboring adult females (0.4%) was minimal. However, the ranges of certain adult males and females overlapped considerably. Direct social interactions were essentially limited to individuals that shared home ranges. Therefore, spatial boundaries coincided with social boundaries (*sensu* Schülke and Kappeler 2003), and pairs of adult males and females can be regarded as the prevailing social unit of *Lepilemur leucopus*.

Currently 24 species of the genus *Lepilemur* are recognized (Ramaromilanto *et al.* 2009) but only a few have been studied in any detail so far. Two of them, *Lepilemur ruficaudatus* and *L. edwardsi*, have been described as pair-living based on spatiotemporal overlap of individual home ranges (Méndez-Cárdenas and Zimmermann 2009; Zinner *et al*. 2003). Mean overlap of 95% KHRs between pair partners was 61% from the male’s and 89% from the female’s perspective, whereas mean overlap between neighboring males was 2.3% and 1.8% between neighboring females in *Lepilemur ruficaudatus* (Hilgartner *et al*. 2012). In *Lepilemur edwardsi*, mean overlap of minimum convex polygons (MCPs) between pair partners was 72% from the male’s and 87% from the female’s perspective (Méndez-Cárdenas and Zimmermann 2009), whereas median overlap between neighboring males was up to 4.9% and up to 6.6% between neighboring females (Rasoloharijaona *et al*. 2006). Thus, all three *Lepilemur* species studied in detail so far exhibit a pair-living social organization.

Other nocturnal primates vary greatly in patterns of male and female spatial distribution. Home ranges of males show mutual overlap and also overlap with the ranges of several females, whereas female home ranges never do so in the aye-aye (*Daubentonia madagascariensis*: Sterling and Richard 1995). In contrast, home ranges overlap within and between the sexes in mouse lemurs (*Microcebus* spp.: Dammhahn and Kappeler 2009; Eberle and Kappeler 2002; Radespiel 2000), hairy-eared dwarf lemurs (*Allocebus trichotis*: Biebouw 2009), giant mouse lemurs (*Mirza coquereli*: Kappeler 1997), greater galagos (*Otolemur garnettii*: Nash and Harcourt 1986), and slender lorises (*Loris lydekkerianus*: Nekaris 2003). In addition, pair-living has been described for several other nocturnal primate taxa, including fork-marked lemurs (*Phaner pallescens*: Schülke and Kappeler 2003), dwarf lemurs (*Cheirogaleus medius*: Fietz 1999), dwarf galagos (*Galagoides zanzibaricus*: Nash and Harcourt 1986), pottos (*Perodicticus potto*: Pimley *et al.* 2005), slow lorises (*Nycticebus coucang*: Wiens and Zitzmann 2003), some tarsiers (*Tarsius* spp.: Driller *et al*. 2009; Gursky-Doyen 2010), woolly lemurs (*Avahi* spp.: Harcourt 1991; Norscia and Borgognini-Tarli 2008), and owl monkeys (*Aotus* spp.: Fernandez-Duque 2007). Thus, in terms of ranging patterns, *Lepilemur leucopus* do not differ fundamentally from other nocturnal primates.

### Sex-Specific Ranging Behavior

The fact that males ranged over substantially larger areas than females (95% kernel: 0.33 ha vs. 0.18 ha) suggests polygynous tendencies of males. According to Schubert *et al.* (2009), large home ranges allow males to assess the reproductive status of neighboring females and to monitor the presence of neighboring males. Therefore, male *Lepilemur leucopus* may follow a mixed reproductive strategy of maintaining a pair bond while seeking extra-pair copulations, but paternity tests will be required to test this hypothesis because we did not observe any matings. Male home ranges are also larger than female home ranges in *Phaner pallescens* and *Tupaia tana*, which have high rates of extra-pair paternity (Munshi-South 2007; Schülke *et al*. 2004). Extra-pair copulations were also detected in *Lepilemur ruficaudatus*, where males also have significantly larger ranges (95% kernel: 0.99 ha vs. 0.66 ha; Hilgartner *et al*. 2012). Home range size did not differ between the sexes in *Lepilemur edwardsi* (MCP: 2.13 ha for males and 2.07 ha for females; Méndez-Cárdenas and Zimmermann 2009). However, estimates of home range size based on MCPs encompass areas that individuals may have never used and therefore may not accurately reflect patterns of range use (Schülke and Kappeler 2003).

Although the majority (70%) of social units consisted of pairs, some male *Lepilemur leucopus* were associated with two adult females. Similarly, two out of six males of *Lepilemur ruficaudatus* occupied home ranges that overlapped extensively with those of two females (Zinner *et al.* 2003). However, in *Lepilemur ruficaudatus* these two females associated within a shared home range, making it likely that they represented mother–daughter dyads. In contrast, in *Lepilemur leucopus* the two females had exclusive ranges as they were regularly seen within the range of the associated adult male but never within the range of the other adult female. Further, all of these females were adults because all of them were seen with dependent offspring. In Hladik and Charles-Dominique’s (1974) study of the same population, the largest of four males was associated with five females, whereas the other males were associated with either one or two females. Based on morphometric data obtained during our capture (*unpubl. data*), the two males that were associated with two females each were not the largest males. However, their home ranges were 40% and 52% larger than the mean home range of the other males, indicating that energetic constraints on territory defense are not a proximate cause for pair-living from the male perspective (van Schaik and Dunbar 1990).

### Ecology and Ranging Behavior

Although we studied individuals of *Lepilemur leucopus* in a small spiny forest fragment, a crowding effect on ranging patterns seems unlikely. On the one hand, an inverse relationship between density and patch size is frequently observed owing to crowding effects of fragmentation (Bowers and Matter 1997). However, estimates of population density of *Lepilemur leucopus* at Berenty are much higher for the larger gallery forest (810 individuals/km^2^) than for the spiny forest (200–350 individuals/km^2^; Charles-Dominique and Hladik 1971; Hladik and Charles-Dominique 1974). On the other hand, as population densities increase owing to crowding effects, average home range size can be expected to become smaller (Cristóbal-Azkarate and Arroyo-Rodríguez 2007) and/or home range overlap between neighboring social units tends to increase (Arroyo-Rodriguez and Mandujano 2006). Although no quantitative data on ranging behavior are available for the gallery forest population, higher population densities in the gallery forest may imply that home ranges of *Lepilemur leucopus* are smaller in the gallery than in the spiny forest. In addition, observed home range overlap between neighboring individuals in the spiny forest population was minimal.

### Dynamic Spatial Interactions

Using the random gas model, pair partners of *Lepilemur leucopus* approached each other more often to ≤15 m than expected by chance. Schülke and Kappeler (2003) and Hilgartner *et al*. (2012) also used the random gas model to calculate expected encounter rates in *Phaner pallescens* and *Lepilemur ruficautadus*, respectively, assuming that it defines the far end of interindividual spacing within pairs. The results indicated that pair partners of *Phaner pallescens* approached each other more often than expected by chance and that encounter rates in *Lepilemur ruficaudatus* did not deviate from expected values, which was interpreted as a sign of avoidance. According to the random gas model, spectral tarsiers (*Tarsius spectrum*) living in small family groups were found to spend more time in proximity to other group members than predicted by chance (Gursky 2005).

Hutchinson and Waser (2007) pointed out that the number of expected associations is not given correctly by the random gas model if locational data were collected instantaneously. They proposed a corrected model that is also not affected by variable speed or nonuniform distribution of directions. Using the corrected model, pair partners of *Lepilemur leucopus* approached each other less often to ≤15 m than expected by chance, indicating active avoidance.

Similar results were obtained using Doncaster’s model (Doncaster 1990), which allows testing for differences between pairs. Using this model, five out of seven pair partners of *Lepilemur leucopus* approached each other less often than expected. The individuals of the two remaining pairs moved randomly in relation to each other. These two pairs had the smallest joint home range areas. Thus, the restricted area available to them may not have allowed them to avoid each other to the same extent as the partners of the other pairs.

The results obtained using the three different models for testing cohesiveness between pair partners varied considerably. To make a more direct comparison of cohesiveness among nocturnal, pair-living primates, we compared the actual percentage of time pair partners spend within 10 m and 20 m of one another during their active period (Table IV). Whereas *Aotus* spp. are among the most cohesive nocturnal pair-living primates, association rates are comparatively low for *Phaner* spp. and *Lepilemur* spp.

**Table IV:** Overview of percentage of time males and females of pair living nocturnal primates spent in proximity to each other during their activity period

Species	≤10 m (%)	≤20 m (%)	Reference
*Aouts* spp.	100	100	Wright (1994)
*Tarsius spectrum*	28	40	Gursky (2005)
*Periodictus potto*	?	30	Pimley *et al*. (2005)
*Avahi meridionalis*	?	27	Norscia and Borgognini-Tarli (2008)
*Phaner pallescens*	9 (≤15 m)	23 (≤25 m)	Schülke and Kappeler (2003)
*Lepilemur ruficaudatus*	9	20	Hilgartner *et al.* (2012)
*Lepilemur leucopus*	7	23	This study

### Sleeping Associations

Pair partners of *Lepilemur leucopus* never used the same sleeping tree simultaneously, although they shared some of their sleeping trees on consecutive days. In addition, we observed females actively displacing males from their chosen sleeping tree early in the morning at the end of their active period. *Lepilemur edwardsi* shared sleeping trees on average every second day (Rasoloharijaona *et al*. 2003). Similarly, *Lepilemur ruficaudatus* shared sleeping trees every third to fourth day (Zinner *et al.* 2003), but they always occupied different tree holes within the same tree (R. Hilgartner *pers. comm*.). Our study does not support the observation that males and females of *Lepilemur leucopus* sleep together during the day (Russell 1977). However, we observed females sharing their sleeping tree frequently with their offspring.

Other nocturnal primates also exhibit much variation in the composition and stability of sleeping associations. Mouse lemurs (*Microcebus* spp.: Genin 2010; Radespiel *et al*. 2003; Weidt *et al*. 2004), hairy-eared dwarf lemurs (*Allocebus trichotis*: Biebouw 2009) and slender lorises (*Loris lydekkerianus*: Nekaris 2003) sleep in groups of variable size and composition during the day, whereas in aye-ayes (*Daubentonia madagascariensis*: Sterling and Richard 1995) and giant mouse lemurs (*Mirza coquereli*: Kappeler 1997) adults sleep alone. Among pair-living nocturnal primates fork-marked lemurs (*Phaner pallescens*: Schülke and Kappeler 2003), dwarf lemurs (*Cheirogaleus medius*: Fietz 1999), dwarf galagos (*Galagoides zanzibaricus*: Nash and Harcourt 1986), tarsiers (*Tarsius* spp.: Driller *et al.* 2009; Gursky-Doyen 2010), woolly lemurs (*Avahi* spp.: Harcourt 1991), and owl monkeys (*Aotus* spp.: Fernandez-Duque 2007) regularly sleep together, whereas pottos (*Perodicticus potto*: Pimley *et al*. 2005) and slow lorises (*Nycticebus coucang*: Wiens and Zitzmann 2003) rarely do so. In conclusion, considerable variation exists within nocturnal primates with regard to cohesiveness, as measured by the frequency of sleeping associations, and *Lepilemur leucopus* appears to be among the least cohesive species.

### Social Interactions

Although males and females were found to associate in pairs, their rate of social interactions was very low (0.14 interactions/h) and most of their interactions consisted of “sitting within 1-5 m.” Similar low interaction rates were described for *Lepilemur ruficaudatus* (0.27/h; Hilgartner *et al*. 2012) and *Nycticebus coucang*, where social interactions made up only 3% of the activity period (Wiens and Zietzmann 2003). However, rates of agonistic interactions were more than 10 times lower in *Lepilemur leucopus* than in *Phaner pallescens*, with 0.03 compared to 0.48 interactions/h, perhaps reflecting the fact that they compete over qualitatively different nutritional resources, i.e., leaves vs. tree exudates (Schülke and Kappeler 2003). Further, whereas affiliative interactions were exchanged with a rate of 0.22 interactions/h in *Phaner pallescens*, they were virtually absent in *Lepilemur leucopus*. Similarly, affiliative interactions between pair partners were also only very rarely observed in *Lepilemur ruficaudatus* (Hilgartner *et al*. 2012). In contrast, pair partners of *Perodicticus potto* engaged in affiliative behavior during 30% of observations and they exhibited no agonistic interactions (Pimley *et al*. 2005). Rates of aggression were also much lower than the rates of affiliation in cohesive pair-living *Avahi occidentalis* (Ramanankirahina *et al*. 2011).

Rates of direct social interactions are also low in solitary nocturnal primates. For example, in *Mirza coquereli*, affiliative interactions were generally rare and in particular between the sexes, whereas agonistic interactions occurred disproportionately often between the sexes (Kappeler 1997). In *Microcebus murinus*, of the 0.12 social interactions/h more agonistic interactions occurred between nonsleeping group members and more affiliative ones between sleeping group members (Dammhahn and Kappeler 2009). Thus, solitary nocturnal primates and those living in dispersed pairs exhibit similarly low rates of social interactions, with *Lepilemur leucopus* being at the low end of observed values.

Low rates of social interactions do not necessarily indicate a lack of interaction between individuals. Instead, individuals may regulate their relationships mainly through vocal and olfactory signals (Charles-Dominique 1977). In this context, nocturnal primates use loud calls for sexual advertisement (Zimmermann and Lerch 1993) as well as for group aggregation and coordination (Braune *et al*. 2005). *Lepilemur edwardsi* also uses duets to regulate space use and cohesiveness (Rasoloharijaona *et al*. 2006), whereas adult *L. ruficaudatus* rarely coordinate vocal interactions and loud calling basically serves to signal an animal’s presence in its territory and to regulate spacing among conspecifics (Fichtel and Hilgartner 2012). *Lepilemur leucopus* produced five types of loud calls, whose functions need to be studied with future playback experiments. Olfactory sensitivity and acuity is higher for species living in dispersed pairs, compared to those living in cohesive pairs or groups (Barton 2006). Scent-marking behavior is less well developed in *Lepilemur* than in other lemurs because they do not have scent glands, with the exception of paired glands behind the scrotum in males (Petter *et al.* 1977; Schilling 1979). *Lepilemur mustelinus* uses nonnutritive tree gouging as a marking behavior to display ownership of sleeping sites whereas the same behavior is absent in *L. edwardsi* (Rasoloharijaona *et al*. 2010). Marking behavior in *Lepilemur leucopus* is inconspicuous; however, we occasionally observed males placing scent marks by rubbing their anogenital region against tree trunks, and only males performed branch bashing displays. Thus, also in terms of communicative complexity, *Lepilemur leucopus* ranges near the low end of among primates (McComb and Semple 2005).

### Possible Causes of Pair Living

Given the virtual absence of direct male–female association and interaction, it is intriguing to speculate about the possible causes of pair living in this and other species living in dispersed pairs (Schülke 2005). Sportive lemurs are seasonal breeders, with a short mating season around May/June (Hilgartner *et al*. 2008; Randrianambinina *et al.* 2007). The short annual mating season and female spatial distribution seem to limit the potential of males to monopolize more than one female in *Lepilemur ruficaudatus* (Hilgartner *et al*. 2012), and in mammals more generally (Lukas and Clutton-Brock 2013). Thus, mate guarding and female defense may be important components of male reproductive strategies. This is reflected by increased night range overlap between pair partners during the mating season (see also Hilgartner *et al*. 2008). The small female ranges may facilitate monopolization of the ranges of two females for some males. However, male and female *Lepilemur leucopus* occupied mutually overlapping home ranges also outside the short annual mating season. Searching for a new mate every year may be more costly than defending the joint territory year-round because of the energetic costs of roaming, increased predation risk during roaming, and the risk of injuries from intrasexual competition (Ralls *et al*. 2007). Females may potentially profit from year-round associations with a male by territorial defense, and hence reduced food competition (Wrangham 1979), by protection against infanticide (van Schaik and Kappeler 1997) and by paternal care (van Schaik and van Hooff 1983). However, competition for food is low, even during the lean season, indicating that a possible resource defense strategy by males may play only a minor role in this species (*unpubl. data*). Although paternal care is absent in sportive lemurs (Hilgartner *et al*. 2008), infanticide was observed in *Lepilemur edwardsi* (Rasoloharijaona *et al*. 2000) and we observed one case of male infanticide, indicating that infanticide risk may play a role in the evolution and maintenance of dispersed pairs (Opie *et al*. 2013). Further, females may preferentially mate with males they are familiar with (Fisher *et al*. 2003), and the stability of pair bonds may have an effect on long-term reproductive success. In owl monkeys (*Aotus azari*) stable pairs reproduced once a year, whereas only about 20% of newly formed pairs produced offspring within the first year of pair formation (Fernandez-Duque and Huck 2013).

### Social Complexity

On the basis of all currently recognized dimensions of social complexity (Freeberg *et al*. 2012), white-footed sportive lemurs lie at or near the low end of all respective measures. Their modal group size is at the theoretical minimum and they rarely interact with neighbors, i.e., they do not interact frequently with many different individuals. Moreover, observed social interactions with physical contact were limited to bouts of agonism, and neither a single bout of grooming nor mating were observed in >1500 h of observations. In fact, most pair partners actively avoided each other, and most interactions were recorded only because we defined sitting in proximity as a social interaction. Thus, social interactions did also not occur in many different contexts and they occurred with negligible frequencies. Finally, this lack of social complexity was not compensated by high levels of communicative complexity because the sizes of their vocal and olfactory repertoires were among the smallest ones reported for primates so far. Thus, we propose that this species of sportive lemur can be used to define a baseline of primate social complexity against which comparable data from other species can be scaled, so that the adjective “highly social” that is increasingly being used to characterize species (Bateman *et al*. 2012; Hoelzel *et al*. 2007) can actually be used in a meaningful way.

## Conclusions

White-footed sportive lemurs were found to live in dispersed pairs, resolving questions about their social organization based on earlier studies at the same site. Males and females sharing a home range were characterized by low spatial cohesiveness, including signs of active avoidance, as well as very low rates of direct social interactions. This social system may ultimately be the result of male reproductive strategies, but the determination of the possible causes of pair living in this species requires further study. In any event, *Lepilemur leucopus* is the most asocial of all primates living in pairs studied to date, placing it at or near the primate baseline of social complexity.
